# Quercetin-3-Glucoside Extracted from Apple Pomace Induces Cell Cycle Arrest and Apoptosis by Increasing Intracellular ROS Levels

**DOI:** 10.3390/ijms221910749

**Published:** 2021-10-04

**Authors:** Arti Nile, Shivraj Hariram Nile, Juhyun Shin, Gyunseok Park, Jae-Wook Oh

**Affiliations:** 1 Department of Stem Cell and Regenerative Biotechnology, Konkuk University, Seoul 05029, Korea; aartibmahajan@gmail.com (A.N.); junejhs@konkuk.ac.kr (J.S.); bhs2945@hanmail.net (G.P.); 2 Laboratory of Medicinal Plant Biotechnology, College of Pharmacy, Zhejiang Chinese Medical University, Hangzhou 310053, China; nileshivraj@yahoo.com

**Keywords:** apple pomace, quercetin-3-glucoside, apoptosis, ROS generation, cell cycle progression, anticancer

## Abstract

Cervical cancer is a life-threatening disease and the fourth most common cancer among women worldwide. Apple pomace is a multifunctional phenolic compound possessing effective biological activity against cervical cancer cells. This study aimed to investigate the anticancer effects of quercetin-3-glucoside (Q3G) extracted from apple pomace in HeLa cell lines and analyze its molecular mechanisms. High-performance liquid chromatography revealed that Q3G, coumaric acid, phloridzin, quercetin, and phloretin are the major polyphenolic compounds constituting apple pomace. Among them, Q3G possessed the greatest antioxidant and anti-inflammatory effects in vitro and exhibited significant cytotoxic effects in HeLa cells in a dose-and time-dependent manner. Flow cytometric analysis indicated that Q3G induced cell cycle arrest at the S phase in a time-dependent manner by altering cyclin-dependent kinase 2. Moreover, it induced apoptosis via chromosomal DNA degradation and increased reactive oxygen species generation. Furthermore, Q3G treatment altered the apoptosis-associated protein expression in the cells by activating caspase-9/-3, downregulating anti-apoptosis protein B-cell lymphoma (Bcl)-2 expressions and up regulating the pro-apoptotic Bcl-2-associated X protein. BH3-interacting domain death agonist cleavage occurred prior to the degradation of an anti-apoptotic Mu-2-related death-inducing gene involved in cell death signaling. Consequently, apple pomace Q3G holds promise as an anti-inflammatory and anticancer agent for treating cervical cancer.

## 1. Introduction

Apple (*Malus domestica*), one of the most widely consumed edible fruits worldwide, is considered a major source of bioactive compounds such as phenolics, flavonoids, terpenoids, and carotenoids possessing various health benefits, such as providing a source of antioxidants in the diet and reduction of cancer risk in humans [1,2]. Moreover, a significant amount of apple waste, collectively called pomace, is generated during the industrial processing of apple fruit for the manufacture of various by-products, primarily comprising juices and jams [3]. In recent years, various techniques have been employed in the utilization of apple pomace for the direct extraction of various bioactive compounds—such as polyphenolics—possessing various biological properties. These have received much interest over the years as a natural and low-cost source of phytochemicals [2,4]. These phytochemicals play a significant role in maintaining human health by exercising preventive effects against cardiovascular diseases, diabetes, and cancer [1,5]. Apple pomace, along with its phenolic compounds, was found to be significantly effective against colon adenocarcinoma cells, breast adenocarcinoma cells, as well as cervix epithelioid carcinoma cell lines [6]. Chlorogenic acid, epicatechin, caffeic acid, coumaric acid, quercetin-3-glucoside (Q3G; isoquercetin), quercetin, phloridzin, and phloretin are the major polyphenolics found in apple pomace [7,8].

McCann et al. [9] reported that phenolic compounds extracted from apple waste exhibited promising anticancer properties against HT-29, HT115, and CaCo-2 cell lines. Moreover, polyphenolic antioxidant compounds extracted from apple pomace—primarily chlorogenic acid, phloridzin, epicatechin, and catechin—have proven to be effective in the reduction of cardiovascular dysfunction, diabetes, and cancer [10,11]. In vitro studies on colon cells treated with apple extract phenolic compounds confirmed protection against DNA damage and increased barrier integrity, leading to decreased rates of aggressive mutations associated with tumors as well as decreased risk of colon cancer [2]. Q3G is a naturally occurring polyphenolic compound extracted from apple pomace that possesses promising antioxidant, anticancer, and anti-inflammatory properties. It alleviates ethanol-induced hepatotoxicity, oxidative stress, as well as inflammatory responses via the nuclear factor E2-related factor/antioxidant responsive element (Nrf2/ARE) signaling pathway, substantially reduces ethanol-induced cytotoxicity, and protects hepatic cells against ethanol-induced liver injury [12,13]. Q3G has been shown to differentially suppress epidermal growth factor-induced migration and inhibit the infiltration of pancreatic cancer cells in a dose-dependent manner. Furthermore, it exerts an anti-migratory effect at a relatively low dose compared to other forms of quercetin [14,15].

Although several potential anticancer properties of Q3G have been previously reported in various cancer cell lines, very limited information is available in the literature with regard to the anticancer effects of apple pomace Q3G on HeLa cervical cancer cells. To this end, this study aimed to investigate the anticancer effects of Q3G extracted from apple pomace against HeLa cervical cancer cell lines, by triggering reactive oxygen species (ROS) generation, inducing apoptosis, and inhibiting cell cycle progression, thus establishing Q3G as a novel anticancer agent.

## 2. Results and Discussion

### 2.1. Phenolic Compounds Constituting Apple Pomace

**Figure 1:**
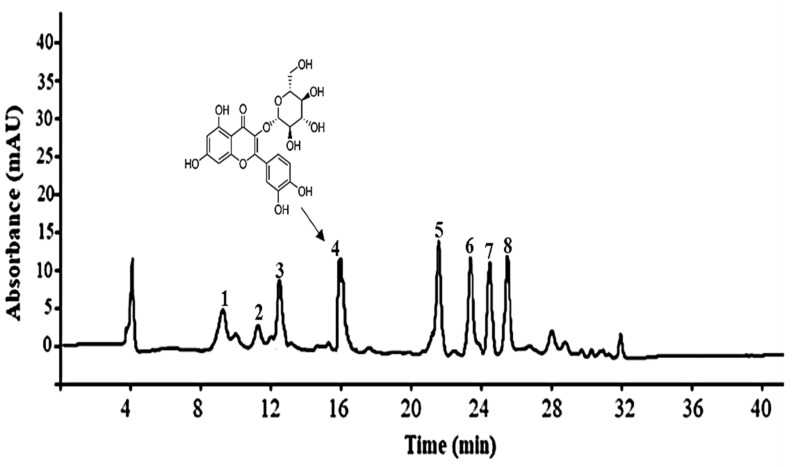
HPLC analysis of polyphenolic compounds extracted from apple pomace and structure of quercetin 3-β-D-glucoside. (1) chlorogenic acid; (2) epicatechin; (3) caffeic acid; (4) quercetin-3-glucoside; (5) coumaric acid; (6) phloridzin; (7) quercetin; (8) phloretin.

The polyphenolic compounds present in apple pomace were analyzed and quantified using HPLC-DAD analysis. The chromatograms of apple pomace extracts obtained by 80% ethanol extraction have been depicted in Figure 1, wherein the concentration is represented as μg/mg dry weight of apple pomace. A total of eight polyphenolic compounds were quantified in apple pomace, as follows: chlorogenic acid (5.2 ± 0.082) epicatechin (2.6 ± 0.056), caffeic acid (3.8 ± 0.036), quercetin-3-glucoside (8.6 ± 0.091), coumaric acid (11.5 ± 0.055), phloridzin (10.2 ± 0.091), quercetin (8.2 ± 0.81), and phloretin (9.8 ± 0.52), μg/mg of dry apple pomace (Table 1). These results are concurrent with the findings of previous studies involving phenolic compound quantification in apple pomace [7,16]. In one such study, 85% methanol was observed to be a more effective solvent for the extraction of apple phenolic compounds, whereby a mixture of solvents and water were more effective solvents for the extraction of phenolic compounds than a mono-organic solvent [1]. Extensive studies have indicated that apple pomace consists of numerous types of polyphenolic compounds that are similar to those observed in apple fruit—including flavanols, flavones, flavanones, chalcones, hydroxycinnamic acids, and anthocyanins [17,18]. The variations in phenolic compound composition and concentration between apple pomace studies could be attributed to the differences arising from varying solvents, the extraction conditions, and the varieties of apples used in industrial processing.

### 2.2. Antioxidant Potential

The antioxidant potential of apple pomace phenolic compounds was measured via commonly employed antioxidant assays: 2,2-Diphenyl-1-picrylhydrazyl (DPPH), ferric reducing antioxidant power (FRAP), 2,2′-azino-bis(3-ethylbenzothiazoline-6-sulfonic acid) (ABTS), and oxygen radical absorbance capacity (ORAC), the results of which have been presented in Table 2. Q3G extracted from apple pomace exhibited the highest radical scavenging activity, with 90.75% (DPPH), 88.22% (FRAP), 82.76% (ABTS), and 75.29% (ORAC) inhibition, respectively. It was followed by quercetin > chlorogenic acid > coumaric acid > caffeic acid > epicatechin > phloretin > phloridzin, when compared to ascorbic acid, which exhibited 92.12% (DPPH), 87.69% (FRAP), 80.16% (ABTS), 75.32% (ORAC) inhibition, respectively, as standard antioxidant agents (Table 2). Several previous studies have reported the antioxidant effects of polyphenolics extracted from apple pomace, and our results were concurrent with the same [7,19,20]. These phenolic compounds extracted from apple pomace have been previously reported for their antioxidant activity and found to be most effective in scavenging free radicals [7,20,21,22].

### 2.3. Anti-Inflammatory Effect

Among all the apple pomace phenolic compounds, Q3G was identified to be the most active towards cyclooxygenase (COX)-1 (3.62), COX-2 (5.66) and lipoxygenase (LOX-5) (2.31), half maximal inhibitory concentration (IC_50_) in μg/mL. Moderate efficacy was observed with quercetin and coumaric acid, while the rest of the phenolic compounds exhibited only a low inhibitory effect against COX-1, COX-2, and LOX-5 (Table 2). In this respect, indomethacin inhibited COX-1 and COX-2 with the IC_50_ values at 1.25 and 1.38 μg/mL, respectively, whereas zileuton inhibited LOX-5 with the IC_50_ values at 1.14 μg/mL. COX-1/-2 and 5-LOX are important enzymes involved in the inflammatory progression pathways in mammalian cells, signifying them to be of particular clinical relevance [23]. COX-1 and COX-2 possess similar functions in catalyzing the target for drug development for inflammation, inflammatory diseases, certain vascular diseases, and cancers [24]. Inflammatory and pathogenic conditions activate the enzymes COXs and 5-LOX, the key enzymes in the synthesis of prostanoid and eicosanoids from polyunsaturated fatty acids (PUFAs), which are involved in various inflammatory and allergic disorders. Combination therapeutic agents that block both COX2 and 5-LOX are preferred for inhibiting arthritic inflammation by minimizing any adverse effects [25].

### 2.4. Dose- and Time-Dependent Cytotoxicity of Q3G

**Figure 2:**
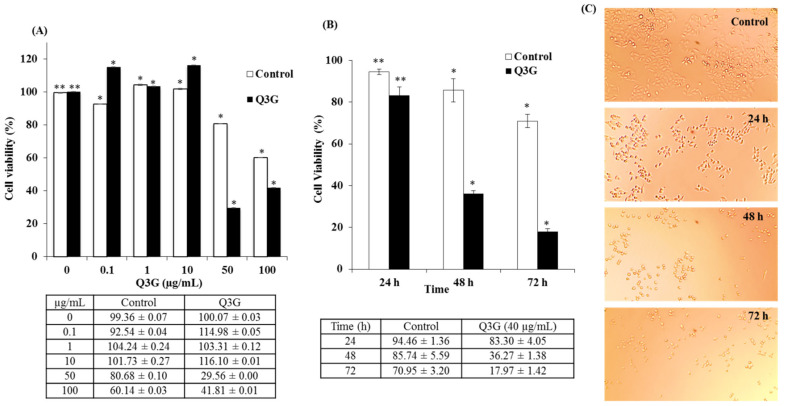
WST assay results for Q3G cytotoxicity in Hela cells: (**A**) Dose dependent cytotoxicity: Cells were treated with different concentrations of Q3G for 48 h. (**B**) Time dependent cytotoxicity: Cells were treated with 40 µg/mL of Q3G for 24, 48, and 72 h. Data in the table are mean ± SD of two independent experiments in triplicate. (**C**) Phase-contrast images showing the morphological changes in HeLa cells documented after 24, 48 and 72 h of incubation with or without Q3G. * *p* < 0.05, ** *p* < 0.01 indicate the significant differences from the control.

Quercetin is one of the most prevalent flavonoids extracted from natural products, and many reports have described its promising anticancer effects in various cancer cell lines [26,27]. However, there is limited research addressing the anticancer effects of quercetin derivatives, especially quercetin glucosides. Of these metabolites, quercetin-3-sulfate (Q3′S) and Q3G constitute a very important class of quercetins and exhibit highly varied biological properties compared to the original compounds [28]. Therefore, it is critical to assess their anticancer activities. To this end, the present study tested Q3G as an anticancer compound. The effect of Q3G on the viability of HeLa cells was determined by treating the cells with different concentrations of Q3G and verifying Q3G-induced cell toxicity via a 2-(4-iodophenyl)-3-(4-nitrophenyl)-5-(2,4-disulfophenyl)-2H-tetrazolium- monosodium salt-1 (WST) assay. HeLa cells treated with increasing concentrations of Q3G (0–100 µg) for 48 h demonstrated significant growth inhibition at concentrations higher than 10 µg/mL, clearly indicating that Q3G exhibited cytotoxicity in a dose-dependent manner. Furthermore, quercetin inhibited the viability by 50% (IC_50_) at 40 µg after 48 h of treatment (Figure 2A). These results suggest that Q3G exerted a significant inhibitory effect on the growth of HeLa cells compared to the untreated controls. Additionally, the decrease in cell viability after 48 h was more significant than that after 24 h. Q3G (40 µg) treatment reduced viability by 83%, 36%, and 18% after 24, 48, and 72 h, respectively (Figure 2B). Moreover, microscopic examination of the cells treated with Q3G at various time points in comparison with the untreated control exhibited the characteristic rounding of the dying cells (Figure 2C). Among the main flavonol glycosides from natural sources, in particular quercetin-3-glucoside (Q3G), considered to be the main bioactive compound responsible for various biological activities [29]. Interestingly, it was found that the Q3G induces MCF-7 apoptosis and necrosis, so studying the anti-cancer activity of Q3G is an interesting phenomenon that can be used to develop Q3G as an effective natural bioactive agent in cancer treatment [30]. Yang and Liu (2009) used in their study a pure form of Q3G in combination with apple extract and showed the anti-proliferative activity against growth of MCF-7 human breast cancer cells [31]. They found that the combination of apple extract and Q3G significantly improved anti-proliferative activity towards the growth of MCF-7 human breast cancer cells in vitro compared to apple extract and Q3G alone. As far as we know, there has been little research on Q3G as an anticancer against various cancer lines.

### 2.5. Q3G Treatment Regulated the Cell Cycle Arrest

**Figure 3:**
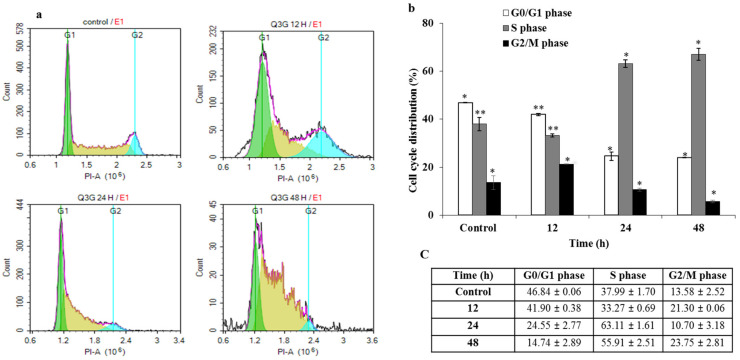
Hela cell cycle analysis with PI: (**a**): After treatment with 30 µg of Q3G for 24 or 48 h, HELA cells were analyzed for cell cycle distribution with Novocyte flow cytometer. The representative plot shows cells in G_0_/G_1_ phase (green), S phase (yellow), and G_2_/M phase (blue). Compared to normal DMSO treated cells (Control), Q3G treated cells were arrested at S phase and G_2_/M phase. (**b**): Graph shows the percentages of cells in G_0_/G, S and G_2_/M phase. (**c**): Data in the table are mean ± SD of two independent experiments in triplicate. * *p* < 0.05, ** *p* < 0.01 indicate the significant differences from the control.

**Figure 4:**
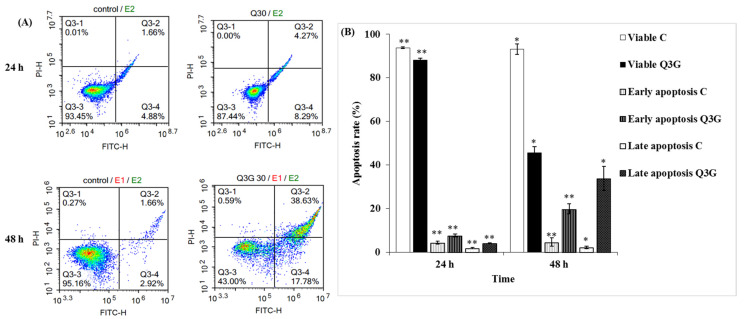
Q3G induces time-dependent apoptosis in Hela cells. (**A**) PI and FITC-Annexin V co-staining identified cell death of Hela cells with or without 30 µg Q3G treatment analyzed by flow cytometry. (**B**) Graph shows the percentages of apoptotic cells. Experiments were performed in triplicate, and the results were expressed as mean ± SD. C: Control, DMSO: dimethyl sulfoxide, PI: propidium iodide, FITC: fluorescein isothiocyanate, Q3G: Quercetin 3-β-D-glucoside. Significantly different from control group, * *p* < 0.05, ** *p* < 0.01.

The inhibitory effect of Q3G on the proliferation of HeLa cells may affect two key cellular mechanisms: cell-cycle arrest and induction of apoptosis. To determine whether Q3G reduced the percentage of viable cells via cell cycle arrest, the distribution of cells in different phases was examined after 12, 24, and 48 h of stimulation with Q3G (30 μg/mL). Cells with DNA content were designated as being in the G_0_/G_1_, S, or G_2_/M phase of the cell cycle. The number of cells in each phase of the cell cycle was expressed as a percentage of the total number of cells examined. As depicted in Figure 3a,b, the number of S phase profiles for HeLa cells was significantly increased, whereas the number of cells in the G_0_/G_1_ phase was significantly decreased after treatment with Q3G. Previous studies have indicated that, depending on the cancer cell type, quercetin can induce cell cycle arrest not only in the G_2_/M phase [32] or S phase [33,34] but also in the G_1_/S phase [35]. It was observed that the proportion of S phase was approximately 33%, 56%, and 63% (Figure 3c), respectively, after treatment with Q3G for 12, 24, and 48 h. In the control group, the frequency of the S cell cycle phase was approximately 38%. Thus, Q3G caused a remarkable increase in the number of S phases in a time-dependent manner. In this study, Q3G prevented HeLa cells from entering the G_2_ phase, resulting in the accumulation of cells in the S phase. This explains why cell cycle regulation is dependent on the type of treatment as well as the cell type. Moreover, the activation of the cyclin E/CDK2 complex in the nucleus drives the progression from the G_2_ to M phase of the cell cycle [36]. S phase arrest in HeLa cells was accompanied by alterations in the cell cycle-regulated protein, CDK2. To this end, Q3G effected S phase arrest by decreasing the protein expression of CDK2 protein (Figure 4A).

### 2.6. Q3G Induced Apoptosis in HeLa Cervical Cancer Cells

Apoptosis occurs as a defense mechanism, such as during an immune response, when cells are damaged by noxious agents or a disease [37]. During the induction of apoptosis, cells undergo a variety of morphological changes—including cellular shrinkage and external exposure of phosphatidylserine on the cytoplasmic membrane—while necrosis is accompanied by swelling of cells and dilation of organelles, resulting in rupturing of the plasma membrane [38]. Early apoptotic cells are Annexin V-positive and PI-negative (Annexin V-FITC^+^/PI^−^), whereas late apoptotic cells are annexin V/PI double-positive (Annexin V-FITC^+^/PI^+^) [39]. In this context, to elucidate whether Q3G-induced enhancement of the S phase cell arrest in HeLa cells was caused by apoptosis or apoptosis accompanying necrosis, the cells were analyzed via flow cytometry using Annexin V-fluorescein isothiocyanate (FITC) and PI staining. Viable cells remained unstained (Annexin V-FITC^−^/PI^−^), whereas early apoptotic cells exhibited Annexin V-FITC^+^/PI^−^ staining patterns and late apoptotic cells exhibited Annexin V-FITC^+^/PI^+^ staining patterns due to a loss of plasma membrane integrity [39]. In DMSO-treated control cultures, approximately 94–95% viable cells were observed. No significant difference in apoptosis (early and late phase) was observed after 24 h of Q3G treatment, when compared with the control cells (Figure 4A). However, after 48 h of exposure to Q3G, a significant increase in apoptotic cells was distinguishable in the HeLa cell population. The results exhibited a 16–22% increase in the number of apoptotic cells (Annexin V-positive cells) after 48 h, compared to that after 24 h of Q3G treatment. Moreover, cell death was observed after 24 h of Q3G treatment compared to that in the control cells (Figure 4A,B). The obtained results are consistent with the WST results, as treatment with Q3G quercetin for 24 h did not significantly affect cell viability and apoptosis. However, cell viability decreased after 48 h of Q3G treatment, and the population of apoptotic cells increased significantly. Approximately 20% of the cells in early apoptosis and 35% in the late apoptosis phase were observed after 48 h of 30 μg/mL Q3G treatment. These results clearly suggest that the induction of apoptosis by Q3G occurred in HeLa cells over a definite period. Further experiments were performed using a dose of 30 μg/mL of Q3G.

### 2.7. Q3G Treatment Altered the Apoptosis-Associated Protein Expression in HeLa Cells

**Figure 5:**
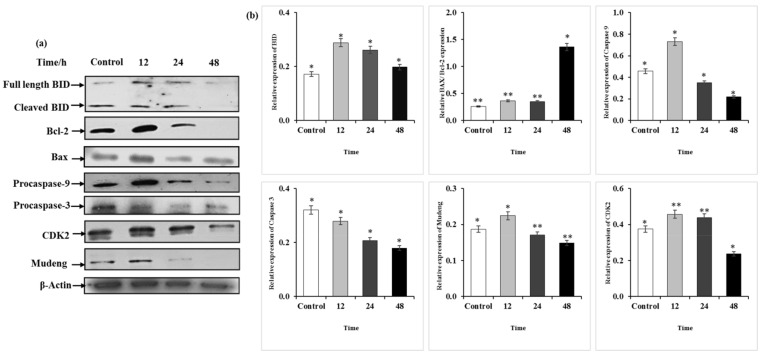
Protein expression of Hela cells treated with Q3G. (**a**): Representative western blots of BID, Bcl-2, Bax, Caspase-3, Caspase-9, Cdk2, Mudeng, and β-actin. (**b**): Quantitative analysis of the expression in comparison to β-actin, an internal control. * *p* < 0.05, ** *p* < 0.01 indicate the significant differences from the control.

To clarify whether Q3G treatment induces apoptosis as well as to evaluate alterations in protein expression, western blotting was performed. HeLa cells were treated with Q3G (30 μg/mL) for the indicated time periods (12, 24, and 48 h), and the prepared lysate was analyzed for various apoptotic pathway proteins. The results suggested that Q3G treatment induced apoptosis in HeLa cells via the effects of apoptosis-associated proteins (Figure 5A,B). Q3G is an inducer of apoptosis, increasing sensitivity to apoptosis, while also functioning as a potential antitumor agent against liver cancer [40]. It has been suggested that high Bax to Bcl-2 ratios disrupt mitochondrial membrane potentials and release cytochrome c, which can result in cellular apoptosis [41]. Another study indicated that Bcl-2 preserves the mitochondrial membrane and inhibits the release of internal calcium into the cytoplasm, whereas Bax is processed on the outer mitochondrial membrane and regulates the release of cytochrome c [42]. During apoptosis, Bax undergoes a conformational change at the mitochondrial level, inducing the release of mitochondrial cytochrome c, which further activates caspases in the cytosol [43]. This study demonstrated that the level of anti-apoptotic protein Bcl-2 was reduced following binding to the BH3-domain-only proapoptotic protein Bid as well as its cleavage, while a concomitant increase in the level of Bax—a proapoptotic protein—was also noted. Apoptosis is primarily induced by caspases, a family of cysteine aspartyl-specific proteases [44]. When activated, an initiator caspase, such as caspase-9, cleaves and activates other execution caspases, such as caspase-3 and caspase-7. This induces the degradation of cellular components during apoptosis [45]. The expression of the initiator caspase, caspase-9—which then activates the effector caspase caspase-3 involved in cell death stimulation—was reduced. Activation of downstream effector caspases such as caspase-3/-9 via proteolytic cleavage of the pro-apoptotic Bid protein was observed.

Mu-2-related death-inducing gene (*MUDENG*, *MuD*) is a novel gene that plays an important role in cell death in various tissues [46]. MuD was initially known to be involved in cell death in cytotoxic T cells [47]. A previous study suggested that Bid activation may occur prior to MuD degradation induced by death stimuli, while Bid cleavage can occur prior to MuD activation and plays an important role in MuD′s functional properties in TRAIL-mediated apoptotic signaling [48]. Strikingly, a similar pattern was observed in our results—Bid cleavage occurred prior to MuD activation, while MuD expression, which is involved in cell death signaling, was markedly reduced after Q3G treatment. These findings indicate that due to the altered level of Bcl-2 family proteins, the apoptotic pathway activity was exacerbated in Q3G treated cells, ultimately resulting in the formation of a caspase-activating multiprotein complex called the apoptosome, which accelerates apoptosis.

### 2.8. Q3G Treatment Induced ROS Production in HeLa Cells

**Figure 6:**
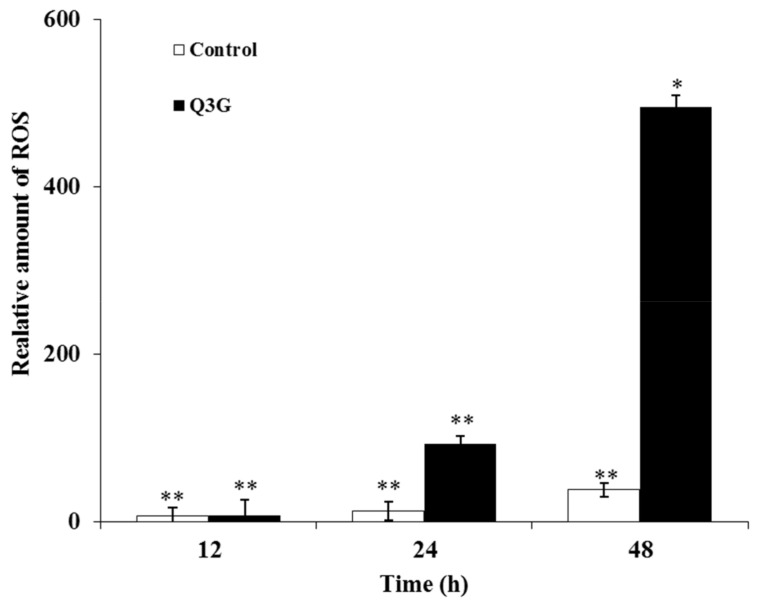
Time-dependent effects of Q3G on the production of intracellular reactive oxygen species (ROS). Hela cells were exposed to 30 µg of Q3G for 12, 24, and 48 h. Amounts of intracellular ROS in Hela cells were quantified using fluorescence spectrometer. * *p* < 0.05, ** *p* < 0.01 indicate the significant differences from the control.

ROS can function as a double-edged sword in regulating cancer survival and cell death. With increased basal oxidative stress, cells become vulnerable to chemotherapeutic agents that further enhance ROS production and weaken the cell’s antioxidant defenses. Conversely, excessive or acute production of ROS can disrupt cell homeostasis through the augmentation of oxidative damage. In this regard, it seems that these ROS-dependent changes could suppress tumor cell growth [49,50]. Additionally, a previous report stated that dietary flavonoids primarily induce apoptosis by increasing intracellular oxidative stress in MCF-7 cancer cells [51]. In order to investigate whether intracellular ROS are implicated in the apoptosis induced by Q3G, the level of ROS in the tested cells was assessed. The results demonstrated that DCFDA fluorescence, which is a standard indicator of cellular ROS generation, was significantly increased following Q3G (30 μg/mL) treatment in a time-dependent manner (12, 24, and 48 h). The results revealed that cells treated with Q3G exhibited high levels of ROS—approximately 6-and 12-fold higher than the control group after 12 and 24 h of Q3G treatment, respectively (Figure 6). The production of ROS is a critical mechanism utilized by anticancer agents against various types of cancer cells [52]. To this end, the results of the present study demonstrated that Q3G may be a potent ROS generator in HeLa cells, disrupting cellular homeostasis in these cells and eventually leading to cell death.

### 2.9. Cytological Changes in HeLa Cells Eventuated by Q3G

**Figure 7:**
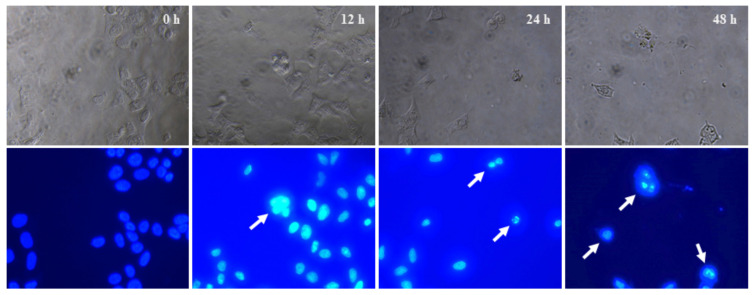
Immunocytochemistry DAPI staining. Effects of Q3G on HeLa cells treated for 12, 24, and 48 h. Control cells showed intact nuclei, whereas all the other groups presented significant visible damage in the nuclei (arrows).

Classical apoptosis is characterized by important morphological features such as membrane blebbing, chromatin condensation, and the formation of apoptotic bodies [53]. Thus, to further confirm whether Q3G decreased the total viable cells through apoptosis of HeLa cells, the cells were incubated with 30 μg/mL of Q3G for 12, 24, and 48 h, followed by 4′,6-Diamidino-2-phenylindole dihydrochloride (DAPI) staining to investigate the formation of chromatin condensation, which was characterized by nuclear fluorescence (white color) (Figure 7). DAPI is a highly specific stain that preferentially binds to the AT region of the dsDNA molecule. Therefore, the DAPI assay is widely used to study DNA damage and chromatin condensation during apoptosis [54]. In addition, previous studies have shown that DNA fragmentation is closely associated with apoptosis [55,56]. The results, as depicted in Figure 7, indicated that the Q3G-treated group exhibited overall cell condensation along with a notable decrease in the number of cells, as compared to the control group (Figure S1). Furthermore, the nuclei of the control group were oval shape with intact and homogenous fluorescence in the DAPI staining, while fluorescent fragments that were segmented into several pieces were observed in the Q3G-treated group. Reductions in the number of cells as well as morphological changes were simultaneously observed. The above results indicated that Q3G inhibited HeLa cell proliferation by inducing DNA damage.

Further studies on targeting several inhibitors or gene silencing/overexpression with sustainable mechanism are currently underway. This study shows the limitations of Q3G’s in vitro study. Therefore, future studies in vivo using animal models are needed to elucidate the underlying mechanisms of the effect of Q3G.

## 3. Materials and Methods

Apple pomace generated during the industrial processing of apples was collected from the apple by-product processing company, Uiseong (Gyeongbuk, Korea). The freshly dried pomace mixed with potassium meta-bisulfite (at 600 ppm) was immediately collected, milled using a grinder [Panasonic Super Mixer Grinder (MX-AC555) Seoul Korea], passed through a 20-mesh (0.84 mm) sieve, and stored at −20 °C until further use as raw material for the extraction process.

### 3.1. Chemicals and Reagents

Q3G, quercetin, epicatechin, chlorogenic acid, caffeic acid, coumaric acid, phloridzin, and phloretin, along with several standards of polyphenolic compounds, were purchased from Sigma-Aldrich (St. Louis, MO, USA), and all chemicals were of HPLC analytical grade with purity higher than 98%. Other materials—including minimum essential medium (MEM), DPPH, ABTS, fetal bovine serum (FBS) (Welgene, Daegu, Korea), and cyclooxygenase (COX)-1/-2 enzymes—were purchased from (Cayman Chemicals, Ann Arbor, WI, USA). In addition, 5-LOX was extracted from potato tubers. Furthermore, WST-1 assay (Dogen, Seoul, Korea), antibiotic solution (Sigma-Aldrich, St. Louis, MO, USA), Gen5 software (BioTek, Winooski, VT, USA), radioimmunoprecipitation assay (RIPA) lysis buffer (Thermo Fisher Scientific, Waltham, MA, USA), Bio-Rad protein assay kit (Bio-Rad, Hercules, CA, USA), anti-caspase-3/-9 antibodies (Abs) (Cell Signaling Technology, Danvers, MA, USA), anti-BH3 interacting-domain death agonist (Bid) Ab, Bcl-2, Bax, anti-cyclin-dependent kinase (CDK)2 Ab (Santa Cruz, CA, USA), anti-mu-2-related death-inducing gene (*MUDENG, MuD*) monoclonal Ab [57], goat anti-mouse IgG and goat anti-rabbit IgG Ab (Jackson ImmunoResearch, West Grove, PA, USA), as well as an enhanced chemiluminescence detection kit (Amersham Pharmacia, Uppsala, Sweden) were also purchased. For the flowcytometric analysis, the following materials were purchased: NovoCyte 1000 benchtop flow cytometer (ACEA biosciences, San Diego, CA, USA), Annexin V-FITC/7- AAD apoptosis detection kit (Sino Biological, Beijing, China), DAPI stain (Sigma St. Louis, MO, USA), and 2′,7′-dichlorodihydrofluorescein diacetate-H2DCFDA (Molecular Probes-Invitrogen Waltham, MA, USA).

### 3.2. Extraction of Phenolic Compounds

For the extraction of phenolic compounds, 5 g of dried apple pomace was extracted with 80% ethanol solution, followed by incubation for 24 h under continuous magnetic stirring at room temperature (RT). The sample was subsequently centrifuged (5210× *g*, 20 min at 4 °C), and the solvent was removed and concentrated at 25 °C using a rotavapor (Rotavapor R 200, Buchi, Switzerland). The resulting residue was stored at −18 °C until further analysis [7,20].

### 3.3. HPLC Analysis of Polyphenolic Compounds from Apple Pomace

Polyphenols present in the apple pomace were identified using a Shimadzu system series LC10Avp (Toyko, Japan) equipped with a diode array detector and Class VP chromatography data station software. Separation of the phenolic compounds was conducted on a reversed-phase Phenomenex C18 (250 × 4.6 µm, i.d. 5 µm) column from Waters (Waters Corp., Milford, MA, USA) protected with a Phenomenex cartridge (C18, 4 mm × 3 mm i.d.) at a temperature of 25 °C. The solvent system consisted of 90% aqueous 0.1% formic acid (solvent A) as well as 10% acetonitrile (solvent B), and separation was carried out using the following gradient: 4–10% B (0–5 min), 10–45% B (5–40 min) with a flow rate of 1 mL/min and injection volume of 10 µL. UV detection was performed at 280 nm wavelength. Quantification was implemented using external standards, with sample runs performed in triplicate, and quercetin as well as Q3G used as reference standards. Identification of phenolic compounds was achieved by comparing the UV profiles and the data with retention time of known standards and extrapolated from the pure quercetin as well as quercetin 3-glucoside standard curves [1,7].

### 3.4. In Vitro Antioxidant Activity

The dose-dependent free radical scavenging effect of apple pomace polyphenolic compounds—including chlorogenic acid, epicatechin, caffeic acid, Q3G, coumaric acid, phloridzin, quercetin, and phloretin (10 μg/mL)—at different concentrations was studied via four radical scavenging assays: DPPH, FRAP, ABTS, and ORAC spectrophotometric assays, as described previously. The percent inhibition was calculated by comparing the optical density of the test samples against the optical density of the blank solution. Ascorbic acid was used as the reference standard [58,59].

### 3.5. In Vitro Anti-Inflammatory Activity

The in vitro anti-inflammatory effect of polyphenolic compounds present in apple pomace—including chlorogenic acid, epicatechin, caffeic acid, Q3G, coumaric acid, phloridzin, quercetin, and phloretin (10 μg/mL)—was studied via COX-1/-2 and 5-LOX enzyme inhibitory assays. The IC_50_ was determined for the studied compounds and reference standards. The in vitro COX-1/-2 enzyme inhibitory activities were assessed using a COX inhibitor screening assay kit (Item No. 560131), according to the guidelines of the manufacturer (Cayman Chemicals, Ann Arbor, WI, USA). Subsequently, the IC_50_ values were calculated for COX-1/-2 activity exhibited by the samples and reference compounds in triplicate. Indomethacin was used as a standard reference enzyme inhibitor in the COX assay. The results were measured at 405 nm, with reference wavelengths of 570 nm and 590 nm, and were expressed as IC_50_ (μg/mL) [25,60]. Furthermore, 5-LOX enzyme was extracted from potato tubers as described previously [61]. The in vitro activity of the enzyme was calculated using a polarographic method connected to a Clark oxygen electrode (Model 782, RC-300; Strathkelvin Instruments, Motherwell, UK), as described previously [25]. The enzyme activity was expressed as moles of oxygen consumed per min/mg protein, as 5-LOX adds oxygen to the substrate, implying that the rate of decrease of oxygen in the reaction mixture was intrinsic to the measurement of enzyme activity. The results were expressed as IC_50_ (μg/mL) for the sample and reference compounds in triplicate. Zileuton was used as a standard reference enzyme inhibitor for the 5-LOX assay.

### 3.6. Cell Viability Assay

The viability of HeLa cells treated with Q3G (0–100 μg/mL, concentrations) was examined for 24–72 h using a colorimetric WST assay. Briefly, HeLa cells were cultured in a clear-bottom 96-well plate at a density of 9 × 10^3^ cells/well in MEM supplemented with 10% FBS and 1% penicillin-streptomycin in a 5% CO_2_ incubator and incubated for 24 h at 37 °C. The Q3G stock solution was prepared in DMSO and stored at −20 °C. Subsequently, the cells were exposed to various concentrations of Q3G (0–100 μg/mL) over a period of 24–72 h. The cell proliferation reagent WST-1 solution (10 μL/well) was added to the plates, followed by incubation in 5% CO_2_ for 2 h at 37 °C in the dark. Thereafter, the plates were shaken thoroughly for 1 min on a shaker at RT, and the absorbance of the reporter substrate was measured at 450 nm and 600 nm (reference wavelength 650 nm) using a microplate reader coupled with the Gen5 software (BioTek, Winooski, VT, USA) [62].

### 3.7. Assessment of Apoptosis

Annexin V-FITC/propidium iodide (PI) assay was performed as per the manufacturer’s instructions (Sino Biological, Beijing, China). Briefly, HeLa cells (2 × 10^5^ cells/well) in 6-well culture plates were treated with Q3G (30 μg/mL) or with DMSO as a vehicle control for 24 and 48 h. Subsequently, floating as well as adherent cells were collected and washed with PBS. The cells were centrifuged at 400× *g* for 5 min at RT and resuspended in binding buffer containing Annexin V-FITC, along with PI. In each experiment, 1 × 10^4^ cells were analyzed for annexin V-FITC/PI staining after incubation for 20 min at RT in the dark.

### 3.8. Cell Cycle Analysis by Flow Cytometry

Cell cycle analysis was performed in HeLa cells as previously described [62]. Cells were seeded into six-well plates at a density of 2 × 10^5^ cells per well and treated with Q3G (30 μg/mL) for 12–48 h. Thereafter, the cells were harvested, washed with PBS, and fixed at 4 °C for 1 h in a 70% PBS-ethanol solution. Cells were subsequently washed twice with PBS, and 50 μL of a 100 μg/mL stock of DNase-free RNase was added to ensure that only DNA, not RNA, was stained. Cell pellets were stained with a fluorescent probe PI (50 μg/mL) and incubated at RT for 30 min, and then analyzed using a NovoCyte 1000 benchtop flow cytometer (ACEA Biosciences, San Diego, CA, USA).

### 3.9. Western Blotting

HeLa cells (1.5 × 10^6^ cells/well) were seeded in six-well plates and cultured in MEM supplemented with 10% FBS for 24 h. Subsequently, the cells were treated with the indicated amounts of Q3G or DMSO as a vehicle control for 12–48 h. Following this, the cells were collected, and total lysates from treated cells were prepared in RIPA buffer for 15 min. Protein concentration was determined using a Bio-Rad protein assay kit with BSA [63]. Equivalent amounts of protein (50 μg/lane) were separated on a 12% SDS-polyacrylamide gel and then transferred onto polyvinylidene difluoride membranes. Membranes were treated with PBS containing 0.05% Tween 20 and 5% nonfat dry milk to block nonspecific binding at 15–25 °C and were incubated overnight at 4 °C with the following specific primary antibodies: caspase-9/-3, Bax, Bcl-2, Bid, CDK2, and MuD. β-Actin was used as an invariant control for equal loading of total proteins. After incubation with the primary antibody, the membranes were incubated with the appropriate secondary antibodies conjugated to horseradish peroxidase. Finally, immunoreactive bands were visualized using an enhanced chemiluminescence substrate (Bio-Rad).

### 3.10. Chromatin Condensation Assay

Approximately 9 × 10^3^ cells/well in a clear-bottom 96-well plate were treated with Q3G (30 μg/mL) or DMSO as a vehicle control for 12–48 h. Cells in each treatment were individually fixed with 4% formaldehyde for 15 min, followed by cell permeabilization by immersion in 0.2% Triton X-100 for 5 min. Subsequently, the cells were washed with PBS three times and stained with DAPI for nucleic acid condensation, as described previously [64].

### 3.11. Intracellular ROS Production

Cellular ROS production was analyzed as previously described [65] with minor modifications to the original methodology. Briefly, cells were seeded at a density of 1.5 × 10^4^/well in a black, clear bottom 96-well plate. The following day, cells were treated with Q3G (30 μg/mL) or DMSO as a vehicle control for 12–48 h. Forty-five minutes prior to completion of the treatment, 100 µL of 2× H2DCFDA dilution was overlayed in each well and incubated in 5% CO_2_ with DCFDA as well as the compounds for 30 min at 37 °C. The fluorescence in each well was assessed on a microplate reader at the end point in the presence of compounds and DCFDA with Ex/Em = 485/535 nm wavelengths.

### 3.12. Statistical Analysis

Each solvent extract was injected thrice during the HPLC analysis and the average peak area was considered the analyte concentration. Fisher’s least significant difference test was performed to calculate the differences between the mean values and significance levels (*p* < 0.05) using one-way analysis of variance for all biochemical assays.

## 4. Conclusions

The recent increase in the incidence of cancer, combined with the inflated cost of several chemotherapeutic drugs with unexpected side effects, and multidrug resistance towards cancerous cells pose major challenges to cancer treatment. Therefore, there is an urgent demand for the development of safe chemotherapy including plant-based natural drugs as efficacious anticancer agents, as they are easily available and cause minimal side effects. In our study, HPLC analysis of apple pomace revealed it to be a significant source of phenolic compounds showing Q3G only as glucoside in of quercetin. We studied the antioxidant, anti-inflammatory, anti-cancer effects, and potential mechanisms of Q3G in cervical cancer cells (HeLa). Q3G exhibited significant cytotoxic effects on human cervical cancer cells in a dose-/time-dependent manner with potent antioxidant as well as anti-inflammatory effects. Also, the Q3G significantly inhibited the proliferation of HeLa cells via cell cycle arrest, and induced apoptosis through increased ROS generation, disrupting cellular homeostasis in these cells and eventually leading to DNA damage and cell death. We also found that Q3G exhibited activation of caspase-9, caspase-3 as well as down-regulation of the anti-apoptotic protein Bcl-2, along with upregulation of the expression of the pro-apoptotic protein Bax. Bid cleavage occurred prior to the degradation of an anti-apoptotic MuD, which was revealed to be involved in cell death signaling. Through these studies, we determined that Q3G has significant anticancer activity against HeLa cells. Additionally works on targeting various inhibitors or gene silencing/overexpression with sustainable mechanism are currently underway. Future studies in vivo using animal models are needed to elucidate the underlying mechanisms of the effect of Q3G.

## Figures and Tables

**Table 1:** HPLC Quantification and Concentration of Polyphenolics from Apple Pomace.

Phenolic Compounds	Peak	Rt (min)	λ Max (nm)	Amount (μg/mg DW)
Chlorogenic acid	1	9	330	5.2 ± 0.082
Epicatechin	2	11	285	2.6 ± 0.056
Caffeic acid	3	12.5	325	3.8 ± 0.036
Quercetin-3-glucoside	4	16	360	8.6 ± 0.091
Coumaric acid	5	21.2	312	11.5 ± 0.055
Phloridzin	6	23.8	290	10.2 ± 0.091
Quercetin	7	24.2	375	8.2 ± 0.81
Phloretin	8	25.1	290	9.8 ± 0.52

DW: Dry weight, Results are the mean of three parallel measurements (*n* = 3).

**Table 2:** In Vitro Antioxidant and Anti-Inflammatory Activity of Apple Pomace Polyphenolics.

Samples	Radical Scavenging Activity (%)				Enzyme Inhibitory Effect IC_50_ (μg/mL)		
	DPPH	FRAP	ABTS	ORAC	COX-1	COX-2	LOX-5
Chlorogenic acid	81.56 ± 2.24 ^d^	75.82 ± 3.35 ^d^	60.62 ± 1.88 ^d^	58.62 ± 2.33 ^d^	14.08 ± 1.11 ^e^	26.69 ± 1.32 ^e^	9.98 ± 0.94 ^e^
Epicatechin	55.18 ± 1.57 ^g^	52.36 ± 1.11 ^g^	48.98 ± 0.82 ^g^	41.10 ± 0.99 ^g^	18.29 ± 1.13 ^f^	24.13 ± 1.31 ^f^	10.09 ± 1.03 ^f^
Caffeic acid	59.11 ± 1.49 ^f^	55.92 ±1.23 ^f^	50.55 ± 1.62 ^f^	45.11 ± 1.70 ^f^	20.32 ± 1.44 ^g^	30.87 ± 1.56 ^g^	12.26 ± 0.91 ^g^
Quercetin-3-glucoside	90.75 ± 2.81 ^b^	88.22 ± 3.21 ^ab^	82.76 ± 1.94 ^a^	75.29 ± 1.22 ^ab^	3.62 ± 0.84 ^b^	5.66 ± 0.95 ^b^	2.31 ± 0.32 ^b^
Coumaric acid	60.45 ± 1.39 ^e^	58.63 ± 1.64 ^e^	51.99 ± 1.61 ^e^	47.69 ± 1.22 ^e^	9.09 ± 0.88 ^d^	15.3 ± 0.91 ^d^	7.96 ± 0.76 ^d^
Phloridzin	40.13 ± 1.12 ^i^	37.91 ± 0.95 ^i^	32.11 ± 0.88 ^i^	30.11 ± 0.86 ^i^	28.63 ± 1.06 ^i^	36.21 ± 1.33 ^i^	18.98 ± 0.85 ^i^
Quercetin	88.28 ± 3.12 ^c^	85.37 ± 1.10 ^c^	80.22 ± 2.17 ^bc^	70.95 ± 1.88 ^c^	6.11 ± 0.91 ^c^	11.3 ± 1.11 ^c^	5.31 ± 0.86 ^c^
Phloretin	45.62 ± 1.45 ^h^	40.91 ± 1.02 ^h^	36.32 ± 1.31 ^h^	38.12 ± 0.98 ^h^	25.14 ± 1.02 ^h^	33.18 ± 1.81 ^h^	16.86 ± 1.22 ^h^
Ascorbic acid *	92.12 ± 3.15 ^a^	87.69 ± 1.36 ^ab^	80.16 ± 3.24 ^bc^	75.32 ± 1.52 ^ab^	NA	NA	NA
Indomethacin *	NA	NA	NA	NA	1.25 ± 0.15 ^a^	1.38 ± 0.12 ^a^	NA
Zileuton *	NA	NA	NA	NA	NA	NA	1.14 ± 0.21 ^a^

* Reference standard antioxidant agent, NA: Not applicable, Results are the mean ± SD of three parallel measurements (*n* = 3). Different superscript letters (a–i) indicate statistically significant differences among the biological activities (*p* < 0.05).

## Data Availability

Not applicable.

## Supplementary Materials

The following are available online at https://www.mdpi.com/article/10.3390/ijms221910749/s1.
