# Supplementary material for: Quercetin-3-Glucoside Extracted from Apple Pomace Induces Cell Cycle Arrest and Apoptosis by Increasing Intracellular ROS Levels
Source: Int J Mol Sci. 2021 Oct 4;22(19):10749. doi: 10.3390/ijms221910749 (PMC8509831; doi:10.3390/ijms221910749)
Supplement: Supplementary file 1 [file ijms-22-10749-s001.zip › ijms-1376180-supplementary.pdf]

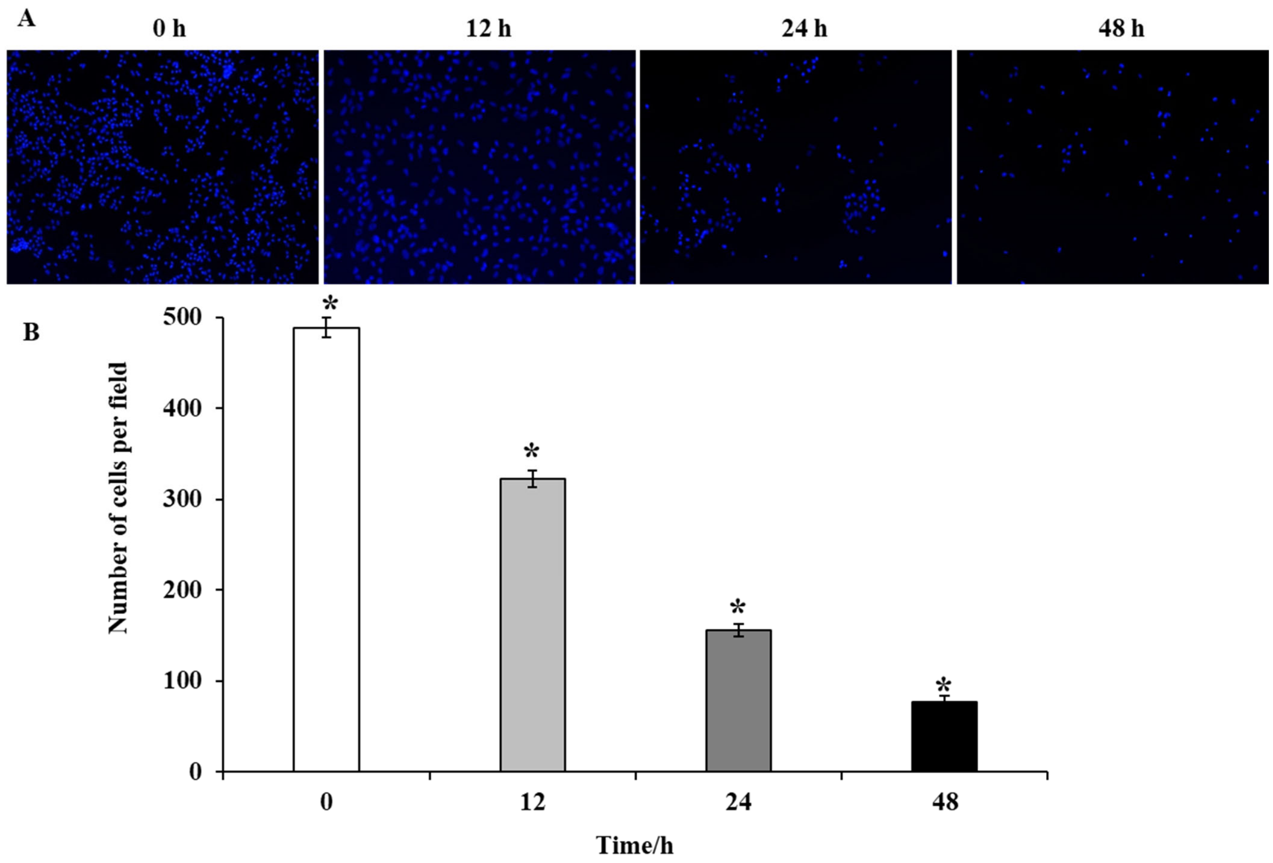

Figure S1. Immunocytochemistry DAPI staining after Q3G treatment. **(A):** Effect of Q3G on Hela cells treated for 0-48 h. **(B):** The total numbers of cells per field were counted and are shown in the graph. Asterisks indicate a statistically significant difference as compared with control (*$P < 0.05$).
